# Numerical Analysis of the Load-Bearing Capacity of a Thin-Walled Perforated Beam Cooperating with Chipboard Panels in a Structural System

**DOI:** 10.3390/ma18102208

**Published:** 2025-05-10

**Authors:** Arkadiusz Denisiewicz, Tomasz Socha, Krzysztof Kula, Wojciech Błażejewski, Marek Wyjadłowski

**Affiliations:** 1 Institute of Building, University of Zielona Góra, ul. Prof. Z. Szafrana 1, 65-246 Zielona Góra, Poland; t.socha@ib.uz.zgora.pl (T.S.); k.kula@ib.uz.zgora.pl (K.K.); 2 Faculty of Mechanical Engineering, Wroclaw University of Science and Technology, Smoluchowskiego 25, 50-370 Wrocław, Poland; wojciech.blazejewski@pwr.edu.pl; 3 Faculty of Civil Engineering, Wroclaw University of Science and Technology, Wyspianskiego 27, 50-370 Wrocław, Poland; marek.wyjadlowski@pwr.edu.pl

**Keywords:** fem, modeling, simulation, thin-walled beams, laboratory tests

## Abstract

This paper presents the results of numerical investigations focused on a structural assembly consisting of thin-walled perforated steel beams joined to a particleboard panel. The simulations were performed using the finite element method (FEM), incorporating both physical and geometric nonlinearities, along with detailed modeling of contact interactions between the beams and panel elements. The primary objective was to establish load-capacity curves for the central beam in structural systems with spans ranging from 3 to 6 m, and to identify failure modes associated with different span lengths. To verify the reliability and accuracy of the numerical approach, laboratory tests were conducted on two representative configurations with spans of 3 and 6 m. Additionally, the mechanical properties of the beam materials were evaluated using samples extracted from the tested elements. The experimental findings confirmed the numerical model’s accuracy and its suitability for analyzing structural responses across the full span range considered.

## 1. Introduction

Cold-formed profile elements play a significant role in modern construction, primarily due to advantages such as low weight, high strength, and design flexibility. Their popularity continues to grow in response to the demand for more efficient, economical, and sustainable building solutions. Cold-formed profiles are particularly known for their high material efficiency [1], requiring less raw material and resulting in lower production and transportation costs. Perforated thin-walled beams have been developed for over a century, with the primary goal of improving the mass-to-stiffness ratio of structural elements while allowing the integration of service systems within the beam depth [2]. Early designs, such as castellated beams with hexagonal openings, evolved into cellular beams with circular perforations, combining improved mechanical performance with architectural aesthetics [2]. Subsequent research introduced new web opening configurations aimed at optimizing both load-bearing capacity and manufacturability [2]. More recently, advances in computational methods have enabled the use of structural topology optimization to design perforated beams with highly efficient opening layouts that surpass traditional cellular and castellated forms [2].

In the United States, initial design recommendations for lightweight structures composed of thin-walled profiles originated from studies conducted at Cornell University, with findings published in 1946 [3]. In the European Union, such structures are typically designed in accordance with Eurocode 3 [4,5,6,7].

Cold-formed elements enable the creation of lightweight yet strong structures capable of supporting significant loads while maintaining low self-weight [6]. Modern construction extensively uses thin-walled profiles in load-bearing systems, particularly in skeletal frames, commercial buildings, and industrial facilities. They are also ideal for high-storage racks, mezzanines, and platforms—key components in the organization of industrial spaces. In addition, cold-formed steel profiles are frequently used in seismic zones due to their ability to deform without losing strength, making them a preferred solution in earthquake-prone regions.

Compared to thicker steel sections, thin-walled profiles are more sensitive to geometric imperfections [8]. Even under relatively low loads, they may exhibit a tendency toward local and global buckling due to their slenderness. This behavior requires special consideration in the design process, particularly under axial and bending loads. Imperfections such as surface irregularities or damage during transport, storage, or assembly must be accounted for in structural analysis [9,10]. This is typically done by introducing initial geometric imperfections based on buckling modes into the computational model [11]. For example, Chinese regulations recommend assuming initial dimensional deviations of approximately L/960 [12].

Given these characteristics, advanced computational methods are used to analyze the behavior of thin-walled structures, capturing deformations, buckling, and interactions between structural modes. Generalized Beam Theory (GBT) is one such method, allowing decomposition of beam deformations into individual modes such as local buckling, global buckling, and torsion [13,14,15,16]. The Semi-Analytical Finite Strip Method (SAFSM) [17] divides the element into strips with simplified displacement models: analytically treated across the width and numerically along the length. A further development, the Constrained Finite Strip Method (cFSM) [18], introduces constraint matrices for isolating specific buckling modes more precisely. Additionally, research highlights the significant influence of the shape and arrangement of web openings on both local and global buckling behavior in perforated beams [19,20].

Despite their advantages, all of the aforementioned methods also have certain limitations. When analyzing elements with perforations, computational challenges may arise. In cases where it is crucial to account for local weakening, stress concentrations, and deformation localization around openings, the widely used Finite Element Method (FEM) can offer more accurate results. Moreover, FEM allows for a relatively straightforward representation of interactions among all structural components within a system and facilitates the estimation of overall load-bearing capacity.

In the present study, FEM was employed to assess the load-bearing capacity of a perforated thin-walled beam integrated into a structural system with a chipboard deck. The same methodology was previously used to successfully evaluate a similar system incorporating a steel grating instead of chipboard [21].

Although numerous studies have addressed perforated beams, their interaction with wood-based deck panels such as chipboard—commonly used in cost-effective structural applications—has not been thoroughly investigated. This study addresses that research gap by numerically and experimentally analyzing the load-bearing behavior of such a hybrid system, providing a validated model and a comprehensive failure analysis across a range of span lengths.

## 2. Materials and Methods

The primary objective of this study was to evaluate the load-bearing capacity of the central beam in a structural system subjected to linear loading transmitted through chipboard decking distributed along the entire beam span. Due to the high cost of laboratory testing and the challenges associated with applying uniform loads experimentally, the research combined laboratory experiments with numerical simulations:

Laboratory tests included:

- Testing a structural system with a span of 3 m, loaded linearly over a length of 1 m;
- Testing a structural system with a span of 6 m, loaded linearly over a length of 1 m;
- Determining the mechanical properties of the steel used for beams and connectors.

Numerical analyses included:

- Creation and validation of numerical models reflecting the tested 3-m span structural system;
- Development and validation of numerical models corresponding to the 6-m span structural system;
- Preparation of numerical models for structures with spans ranging from 3.0 m to 6.0 m (at intervals of 0.5 m), subjected to uniformly distributed loads along their entire lengths;
- Execution of numerical simulations to establish load-bearing capacity curves.

### 2.1. Laboratory Tests

**Figure 1:**
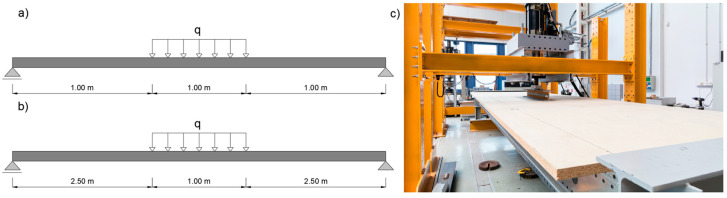
Static loading schemes for beams: (**a**) 3 m beam, (**b**) 6 m beams, and (**c**) testing setup for the 3 m beam.

Laboratory evaluations of the structural assembly and measurements of the actual material properties—specifically the modulus of elasticity and yield strength—were carried out using an INSTRON 8804 testing machine (Norwood, MA, USA). For the structural system tests, a static bending setup was employed, with the load applied over a 1.0 m segment of the beam, as shown in Figure 1.

**Figure 2:**
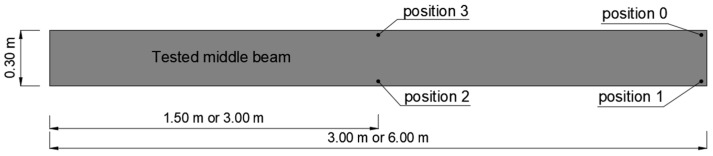
System for measuring horizontal displacements within the elastic deformation range.

Displacement measurement setups were installed at the mid-span and near the connector locations, as shown in Figure 2. Horizontal displacements were recorded using SYLVIAC sensors mounted on the side surfaces of the beams.

The loading procedure included:

- Loading phase: achieving actuator displacement of 7.0 mm within 70 s for the 3 m beam and 15.0 mm within 150 s for the 6 m beams (speed 0.1 mm/s);
- Unloading phase: returning actuator displacement to 0.0 mm within 70 s for the 3 m beam and 150 s for the 6 m beams (speed 0.1 mm/s);
- Elastic testing phase: increasing actuator displacement again from 0.0 mm to the respective maximum displacement values (7.0 mm for the 3 m beam, 15.0 mm for the 6 m beams) while recording displacements and forces to plot deflection-force diagrams;
- Destructive testing phase: after elastic tests, beams were unloaded to an actuator displacement of 0.0 mm, then reloaded until structural failure occurred at a rate of 0.1 mm/s, with deflection–force diagrams recorded throughout.

**Figure 3:**
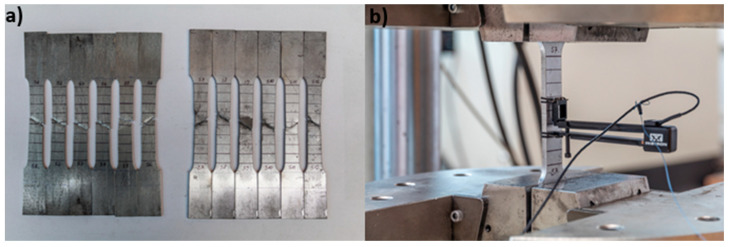
Material samples after strength tests (**a**), and with an extensometer during testing (**b**).

For determining the material strength properties, tensile tests were conducted. An extensometer with a gauge length of 24.1 mm was placed in the pure tension zone, shown in Figure 3. The tensile test procedure was as follows:

- Initial loading: achieving actuator displacement of 0.3 mm within 60 s (speed 0.3 mm/min);
- Unloading: returning actuator displacement to 0.0 mm within 60 s (speed 0.3 mm/min);
- Main loading phase: increasing actuator displacement from 0.0 mm to 0.3 mm over 60 s (speed 0.3 mm/min), during which elongation and force measurements were continuously recorded, resulting in force-elongation diagrams.

Tensile tests were carried out in accordance with the PN-EN ISO 6892-1:2020-05 standard [22] for metallic materials under quasi-static loading conditions. Flat specimens were cut from the flange of the beam, the web, and the connector elements.

### 2.2. Numerical Model

**Figure 4:**
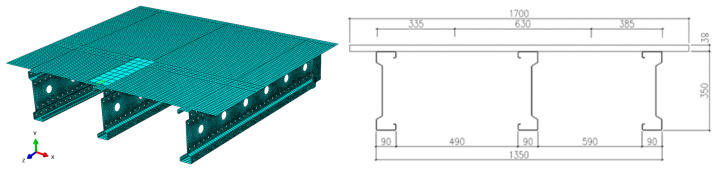
Geometry and discretization of the model. The dimensions are given in mm.

The numerical model was developed using Abaqus Simulia 2022 software [23], based on the finite element method (FEM). The system geometry was entirely represented by shell-type elements and was carefully designed to replicate actual laboratory conditions. The dimensions and relative positions of the components were precisely matched to the physical setup. To improve computational efficiency and reduce simulation time, symmetry was utilized, allowing only half of the structural system to be modeled. The geometric configuration and positioning of elements in the model are shown in Figure 4, which presents one half of the analyzed structure. The beam length was the primary geometric parameter that varied during the simulations.

To model the system geometry, S4R finite elements were used: a 4-node, doubly curved shell element with reduced integration, hourglass control, and finite membrane strains. They are available in the Abaqus 2022 software library [23]. Figure 4 shows the mesh discretization for a 3 m-long beam. An automatic meshing technique based on a progressive front algorithm was employed to generate the finite element mesh. This method began meshing from the model boundaries and expanded inward, ensuring controlled mesh density and high-quality quadrilateral elements, even in areas with complex geometry. Special attention was paid to mesh discretization around the perforations in the beam web. In these areas, the mesh was locally refined to achieve an element size of approximately 3–5 mm, with quadrilateral elements shaped to maintain an aspect ratio close to 1.0. This refinement was necessary to accurately capture local stress concentrations and strain gradients near the hole edges, which are critical to evaluating the structural behavior under loading. The total number of elements varied with beam span:

- 3 m span: 46,558 elements;
- 6 m span: 71,387 elements.

The applied boundary conditions in the model included:

- Connectors were rigidly fixed at the mounting hole locations, with a contact surface diameter of 40 mm.
- A “tie” contact [23] was applied between the connectors and beams, ensuring a fully constrained (rigid) connection.
- Symmetric boundary conditions were imposed along the system’s axis of symmetry, affecting both the chipboard and beams.

Additionally, a “general contact” [23] approach was implemented to define interactions between the bridge deck and beams, with the following parameters:

- Normal behavior; “hard” contact.
- Tangential behavior: Penalty method with a friction coefficient of 0.3.

**Figure 5:**
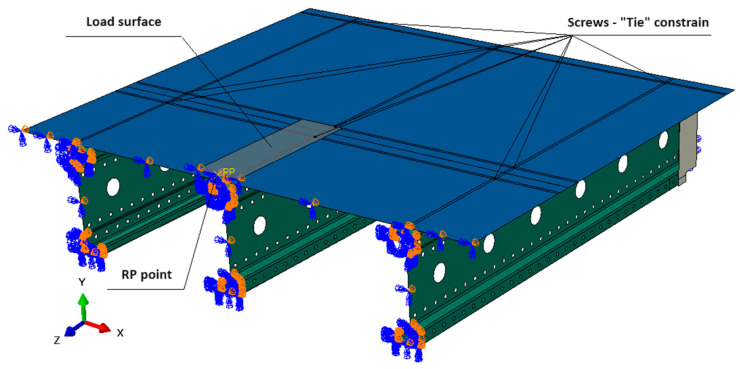
Method of loading—loading surface, screw location.

The deck was modeled as an isotropic plate with actual dimensions. In the numerical model, the deck plates were connected to the central beam at locations corresponding to the screw positions in the physical system. This connection was implemented using a full “tie” constraint applied over an area of 10 × 10 mm. The deck plate model used in the simulation is shown in Figure 5.

The applied load was modeled as a uniformly distributed pressure over a 160 mm-wide surface, replicating the actual test conditions. This load resulted from a concentrated force applied at a reference point (RP), simulating the action of the strength testing machine. The loading method used in the model is illustrated in Figure 5. For the purpose of model validation, the material was assumed to exhibit ideal elastic–plastic behavior, with the following properties:

Beams:

- Young’s modulus E = 205 GPa (determined from laboratory tests).
- Poisson’s ratio ν = 0.3.
- Yield strength R_H_ = 457 MPa (determined from laboratory tests).

Connectors:

- Young’s modulus E = 191 GPa (determined from laboratory tests).
- Poisson’s ratio ν = 0.3.
- Yield strength R_H_ = 362 MPa (determined from laboratory tests).

For the chipboard in strength class P4:

- Young’s modulus E = 2100 MPa, manufacturer’s data.
- Poisson’s ratio ν = 0.3.
- Yield strength R_H_ = 7.5 MPa, manufacturer’s data.

For the chipboard in strength class P6:

- Young’s modulus E = 3100 MPa, manufacturer’s data.
- Poisson’s ratio ν = 0.3.
- Yield strength R_H_ = 11.7 MPa, manufacturer’s data.

Thin-walled beams often exhibit imperfections from manufacturing, assembly, or material properties. Even small deviations can significantly impact structural behavior, particularly buckling sensitivity and nonlinear effects, which are crucial for stability and load-bearing capacity. Incorporating such imperfections into numerical simulations enhances realism and accuracy, supporting structural safety and longer service life. This aligns with modern engineering practices that emphasize realistic performance predictions, especially in safety-critical structures. This study introduced geometric imperfections into the model based on the first buckling mode—a standard method in finite element analysis. In Abaqus 2022, a linear eigenvalue buckling analysis of a perfect model identified the critical load and instability patterns. The first mode was then scaled to 1/300 of the beam length, a typical imperfection magnitude based on industry standards. This scaled mode shape was applied to the original geometry to reflect realistic imperfections. A subsequent geometrically nonlinear analysis allowed assessment of the true structural behavior under load. This revealed effects such as stiffness loss, load redistribution, and failure modes, offering a more reliable performance prediction than linear models.

To verify the mesh independence of the numerical model, a mesh convergence study was conducted. The number of elements was increased and decreased by approximately 25% compared to the base mesh. The results showed that the midspan displacement varied by less than 2%, and the maximum von Mises stresses varied by less than 2.5%, confirming that the adopted mesh density provides reliable and stable results.

### 2.3. Numerical Model for Developing Load-Bearing Curves of the Central Beam

**Figure 6:**
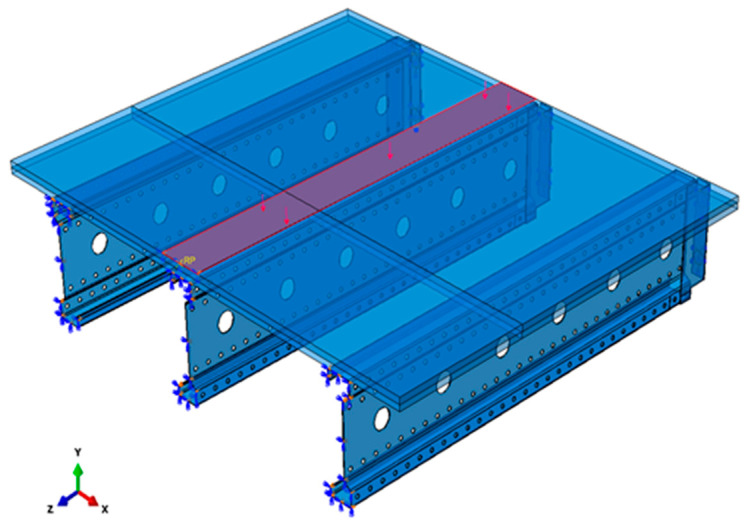
Loading method for targeted numerical analyses.

To determine the load-bearing capacity of a perforated thin-walled beam with a span ranging from 3 to 6 m under continuous loading, a specific modification was introduced in the validated model. In particular, the load application zone was extended across the entire span of the simulated system, as shown in Figure 6.

The load still acts on a strip with a width of 160 mm. This loading distribution eliminates stress concentration that would otherwise occur with linear loading [N/mm] The continuous load *q* acting on the beam [N/mm] can be calculated using the formula *q* = *p* * 160 * *x* where *p* is the surface load [N/mm^2^] applied over a 160 mm-wide band, *x* is a scaling factor representing the proportion of the total load carried by the central beam (e.g., *x* = 0.6 for 60% load distribution, as shown in Table 1). The percentage of load carried by the central beam was determined by comparing deflections. This calculation was based on the condition where LE Max. Principal (Abs) = 5% [23] in the model. LE Max. Principal (Abs) represents the logarithmic strain measure, which is particularly advantageous in nonlinear analyses, where traditional small-strain assumptions are insufficient for accurately capturing material behavior.

## 3. Results and Discussion

This part discusses the outcomes obtained from verifying the numerical model against experimental data, along with findings from the subsequent numerical analyses.

### 3.1. Results of Laboratory Tests

**Figure 7:**
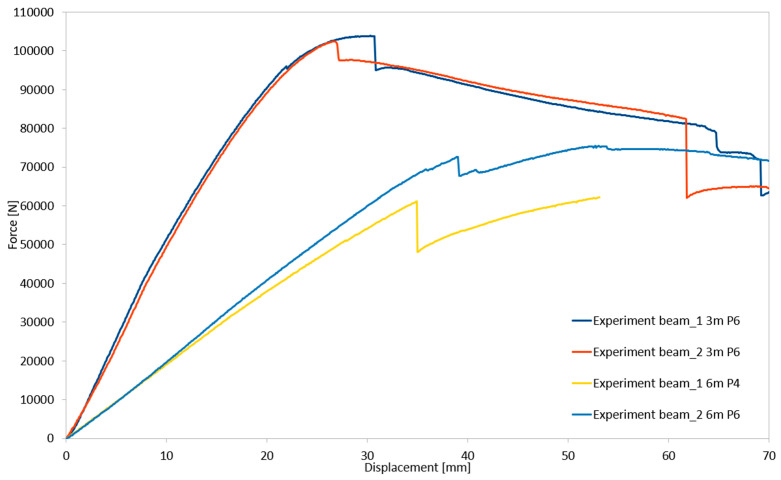
Deflection-force relationships for load-bearing capacity determination in all tested structural systems.

During the laboratory tests, four structural systems were examined: two with a length of 3 m using a P6 chipboard, and one each with a length of 6 m using P6 and P4 chipboards. The load–deflection relationships for all tested configurations, based on their load-bearing capacities, are presented in Figure 7.

Material testing was conducted on a total of 18 samples, with six specimens taken from each of the following components: the top flange, the beam web, and the connectors. The results indicated that the average longitudinal modulus of elasticity was 205.5 GPa for the beam steel and 191.4 GPa for the connector steel, both within the typical range of 200–210 GPa for structural steel. Additionally, the average yield strength was found to be 457.0 MPa for the beam steel and 362.0 MPa for the connector steel.

### 3.2. Results of Numerical Simulations and Model Validation

**Figure 8:**
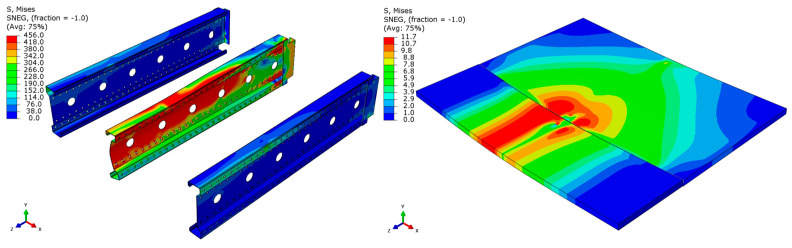
Distribution of reduced stresses for the 3 m perforated beam.

**Figure 9:**
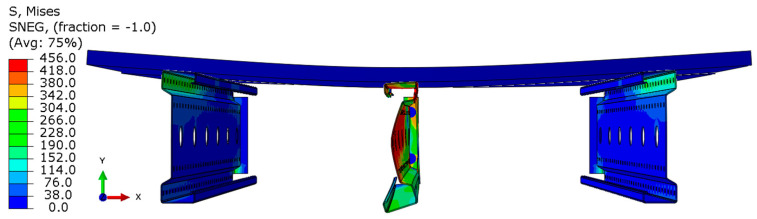
Distribution of reduced stresses for the 3 m perforated beam—cross-section.

**Figure 10:**
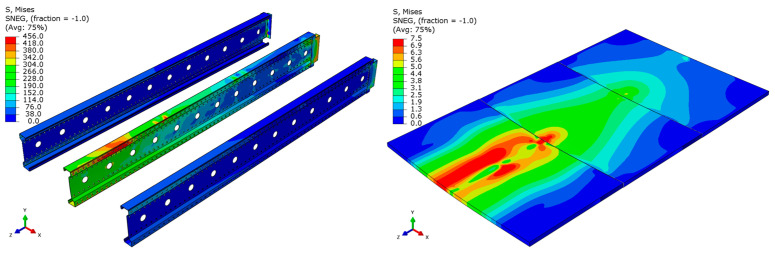
Distribution of reduced stresses for the 6 m beam with P4 deck.

**Figure 11:**
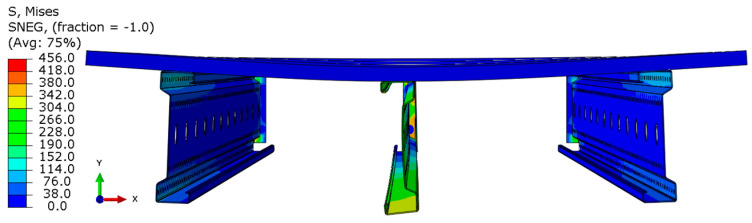
Distribution of reduced stresses for the 6 m beam with P4 deck—cross-section.

**Figure 12:**
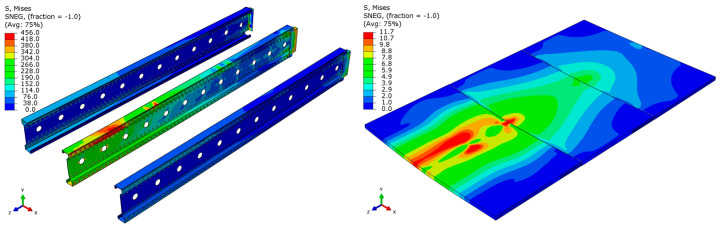
Distribution of reduced stresses for the 6 m beam with P6 deck.

**Figure 13:**
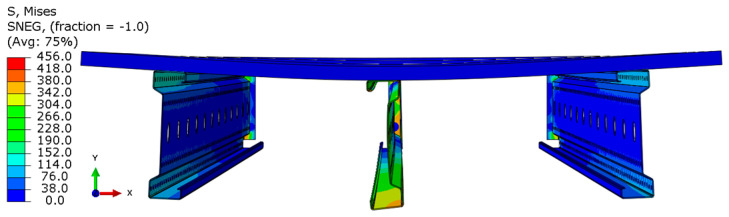
Distribution of reduced stresses for the 6 m beam with P6 deck—cross-section.

Analyzing the stress distributions and deformations presented in Figure 8, Figure 9, Figure 10, Figure 11, Figure 12 and Figure 13, distinct failure mechanisms were observed for the 3 m and 6 m beams:

- For the 3 m beam, the loss of load-bearing capacity occurs due to material plasticization in the central zone of the beam (highlighted in red), as well as at the connection points between the connector and the beam, and within the connector itself. Additionally, failure is contributed to by the fracture of the deck panel.
- For the 6 m beam, a different failure mode is observed. The beam does not exhibit plasticization in either the central or support zones. Instead, the dominant failure mechanism is the fracture of the deck panel (highlighted in red), indicating exhaustion of its bending capacity.
- In both cases (3 m and 6 m beams), connector plasticization is consistently observed, which aligns with the experimental test results.

**Figure 14:**
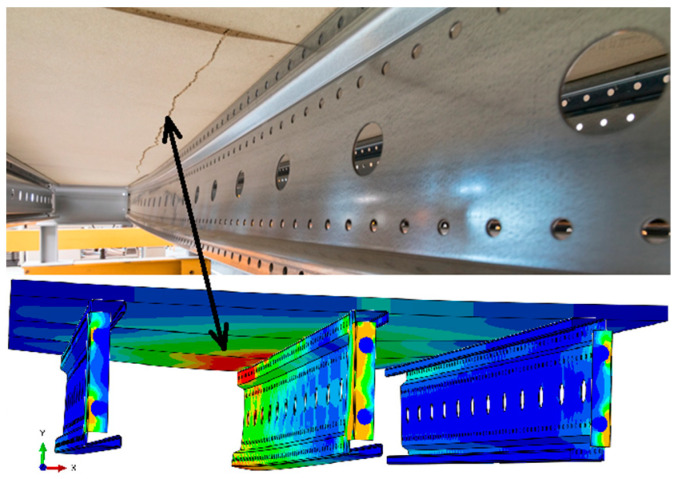
Fracture of the P4 deck panel without significant plasticization of the 6 m beam.

**Figure 15:**
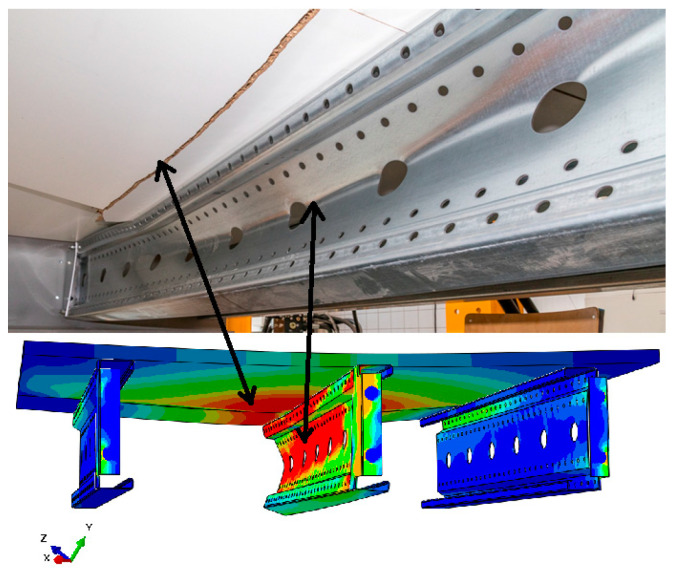
Fracture of the P6 deck panel with significant plasticization of the 3 m beam.

Ensuring the reliability of numerical simulation outcomes requires thorough validation, which helps establish the credibility of results, clarify the model’s limitations, and guide improvements to enhance accuracy and real-world applicability. In this study, model validation was conducted through a combination of qualitative assessments and quantitative analyses:

- Qualitative validation involved assessing whether the structural system’s behavior in the numerical simulations was consistent with the experimental observations.
- The results presented in Figure 14 and Figure 15 illustrate different failure modes, including beam plasticization and deck panel fracture, confirming the consistency between numerical and experimental outcomes.

This behavior is consistent with observations from previous studies [19,20], which highlighted that the increased span length in perforated beams leads to a shift from localized yielding to global deformation mechanisms. Additionally, the stress concentrations observed around the openings confirm earlier findings that careful optimization of the opening shape and arrangement can significantly enhance load distribution and delay failure modes [2].

Quantitative validation was carried out by comparing horizontal displacements and force–displacement curves.

**Figure 16:**
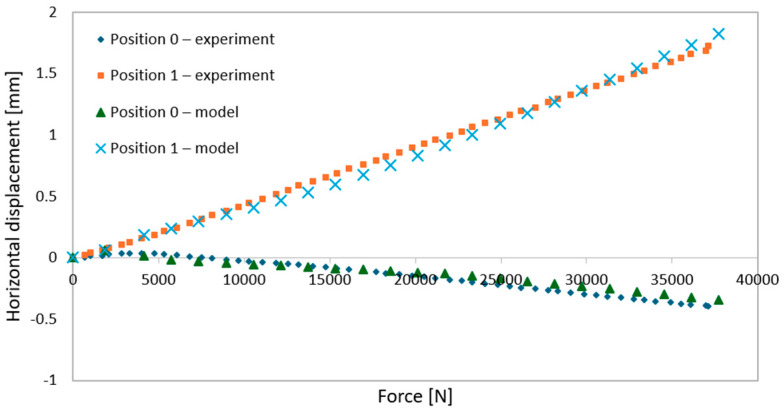
Validation of the model—horizontal displacement of the support cross-section for a 3 m beam.

**Figure 17:**
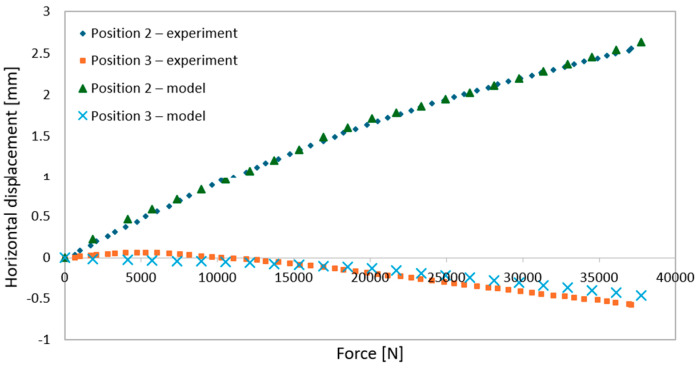
Validation of the model—horizontal displacements of the mid-section for a 3 m beam.

**Figure 18:**
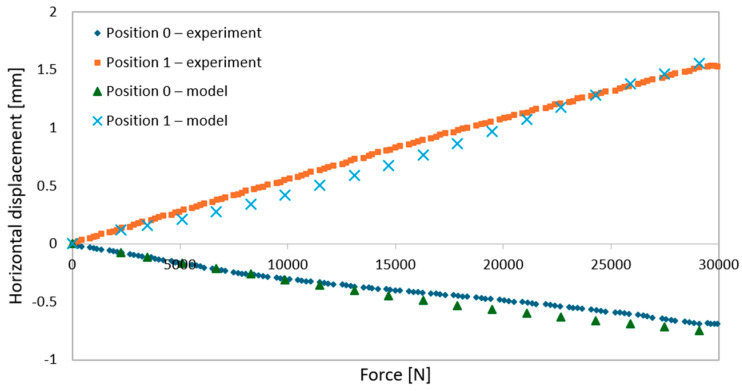
Validation of the model—horizontal displacements of the support cross-section for a 6 m beam with P6 deck.

**Figure 19:**
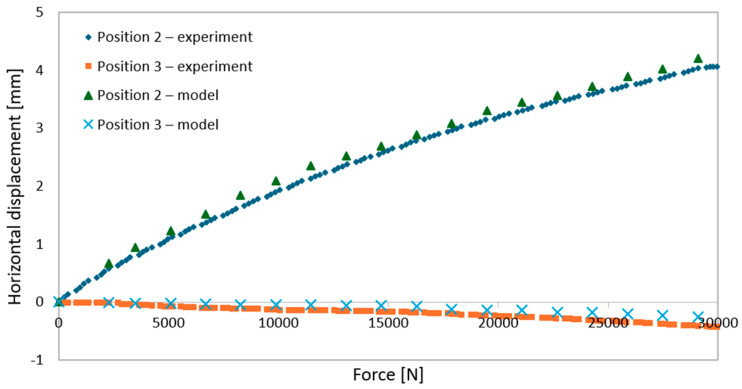
Validation of the model—horizontal displacements of the mid-section for a 6 m beam with P6 deck.

**Figure 20:**
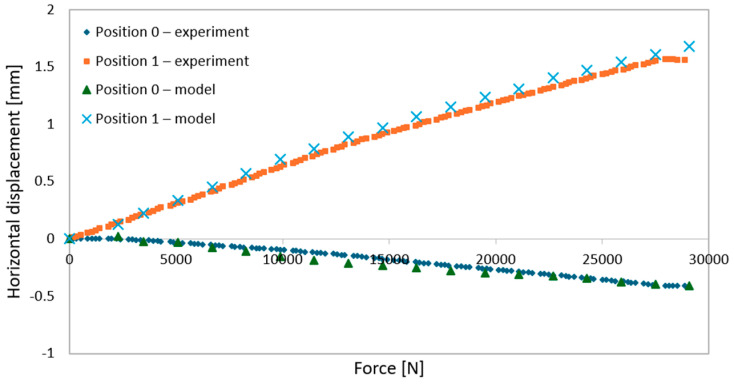
Validation of the model—horizontal displacements of the support cross-section for a 6 m beam with P4 deck.

**Figure 21:**
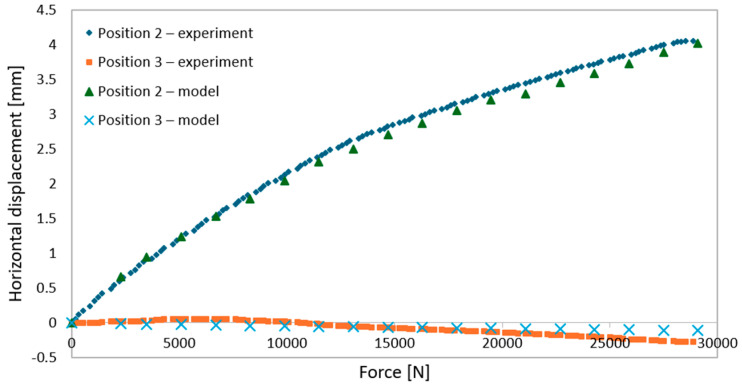
Validation of the model—horizontal displacements of the mid-section for a 6 m beam with P4 deck.

- During elastic-range testing, horizontal displacements were recorded at various measurement points, as shown in Figure 2.
- Figure 16, Figure 17, Figure 18, Figure 19, Figure 20 and Figure 21 present the recorded horizontal displacement values at each measurement point.
- The graphs were generated using average values from all experimental tests conducted within the elastic range of beam behavior and were compared with the results obtained from the numerical model.

**Figure 22:**
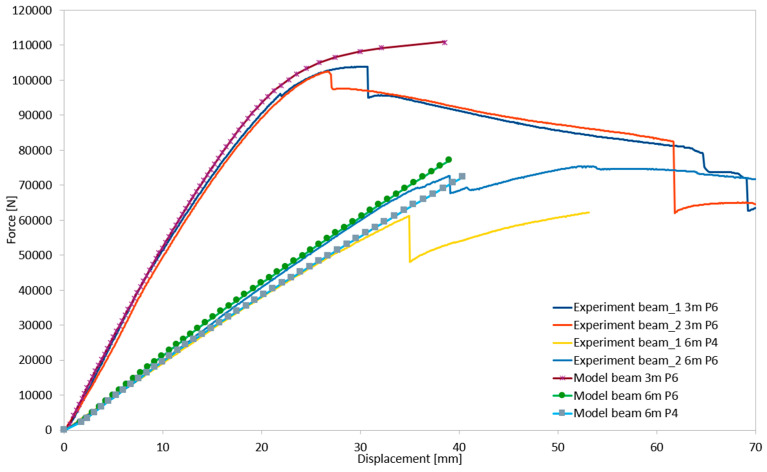
Validation of the numerical model based on laboratory tests.

The final validation of the numerical model’s accuracy was performed by comparing the force–displacement relationships obtained from experimental tests and numerical simulations. The results of this comparison are shown in Figure 22.

The analysis of the results confirms that the proposed numerical model accurately reflects the real behavior of the structural system, offering both precision and consistency. It captures both global and local structural responses, including stress distribution and deformation, while accommodating geometric variations. Its ability to represent structural features such as perforations and joints makes it suitable for a wide range of engineering applications.

The validation of the model builds confidence in its use for advanced simulations, enabling the exploration of various loading scenarios, materials, and structural configurations that would be difficult to investigate experimentally. It also facilitates rapid design prototyping and optimization, ultimately enhancing safety and efficiency.

To quantify the accuracy of the numerical simulations, the maximum load values obtained from experiments and FEM analyses were compared. Table 2 summarizes the peak force values for each configuration:

The differences between numerical and experimental maximum load values remain below 10% for all tested configurations. The best agreement is observed for the 6 m beam with a P4-class deck, while the largest deviation occurs in the 6 m beam with a P6-class deck. These results confirm the high accuracy and reliability of the developed FEM model within the elastic–plastic response range.

Overall, the validated model offers a strong basis for future simulations and engineering work, contributing to more effective and innovative structural solutions.

It should be emphasized that the numerical simulations presented in this study were terminated shortly after the onset of material plasticization. Progressive failure phenomena such as fracture propagation, connector rupture, or large-displacement instability beyond yielding were not included in the model. This approach was intentionally adopted to ensure numerical stability and focus on estimating the maximum load-bearing capacity under quasi-static loading. While this limitation restricts the ability to predict post-peak behavior, the ascending portions of the load–displacement curves match the experimental results with high accuracy, confirming the model’s effectiveness within the elastic–plastic range.

### 3.3. Numerical Simulations of the Analyzed System over the Full Span Range

The objective of the numerical simulations was to estimate the load-bearing capacity of a thin-walled, perforated central beam with a span ranging from 3 to 6 m, functioning within the analyzed structural system under linear loading transferred by a P4-class chipboard deck and uniformly distributed along the entire beam length.

The beam was analyzed under various span lengths to determine how load-bearing capacity and deformation patterns are affected by geometry. The analysis focused on capturing the global bending behavior of the beam, as well as local phenomena such as flange and web buckling near the perforations.

Load-bearing capacity criteria

- The limit values for deflection at the mid-span of the central beam were set at L/250 or L/350 as the load-bearing capacity exhaustion threshold.
- Since laboratory tests and numerical simulations identified the connector as the weakest element in the system, an additional failure criterion was established: reaching 5% LE Max. Principal (Abs) 19 in the connector.
- LE Max. Principal (Abs) represents a logarithmic strain measure, useful in nonlinear analyses where small-strain assumptions are insufficient.

Material properties used in simulations

Beams:

- Young’s modulus: E = 200 GPa
- Poisson’s ratio: ν = 0.3
- Yield strength: R_H_ = 350 MPa

Connectors:

- Young’s modulus: E = 200 GPa
- Poisson’s ratio: ν = 0.3
- Yield strength: R_H_ = 235 MPa

Chipboard (P4-class):

- Young’s modulus: E = 2100 MPa (manufacturer’s data)
- Poisson’s ratio: ν = 0.3
- Yield strength: R_H_ = 7.5 MPa (manufacturer’s data)

The distribution of reduced Mises stresses and system deformation at the final step of numerical analysis is presented in Figure 23, Figure 24, Figure 25, Figure 26, Figure 27, Figure 28, Figure 29 and Figure 30.

Analysis of the results indicates that in all cases, the connector is the weakest component of the system, undergoing plasticization first. This behavior confirms that the connector is critical to the initial load-bearing capacity and plays a significant role in the progressive failure of the structure. Once the connector yields, load redistribution leads to plasticization in the support zones of the beams. These regions are particularly susceptible due to the concentration of internal forces at points of load transfer and restraint.

Plasticization of the support zones ultimately leads to a loss of stability in the mid-span sections of the beams, where bending moments reach their maximum values. As the structure continues to deform under increasing load, the system becomes unstable, indicating the imminent failure of its primary load-carrying components. The final element to fail is the deck panel, which serves as the uppermost structural layer and contributes to the overall stiffness and integrity of the system.

In beams with a span of 3 m, the fracture of the deck panel occurs almost simultaneously with plasticization in the mid-span section of the beam. This suggests a strong interaction between these two components in shorter spans, where structural interdependence is more pronounced. In contrast, for beams with a 6 m span, the deck panel fails significantly earlier. This premature failure is attributed to higher flexural demands and greater deflections in longer spans, which accelerate damage in the deck before full plasticization of the beam’s mid-span occurs.

The failure mechanism evolution observed with increasing span length reflects a shift from local beam plasticization and connector yielding towards earlier fracture of the chipboard deck. This is consistent with literature findings for slender cold-formed members where longer spans amplify deflections and stress redistribution. Additionally, the significant influence of connectors as the critical weakness highlights the need for improved connection detailing. The results suggest that for spans exceeding 5 m, local reinforcements in the deck (e.g., additional plates or ribs) could delay deck failure and enhance system ductility. These findings provide new insights for the structural optimization of lightweight floor systems.

**Figure 31:**
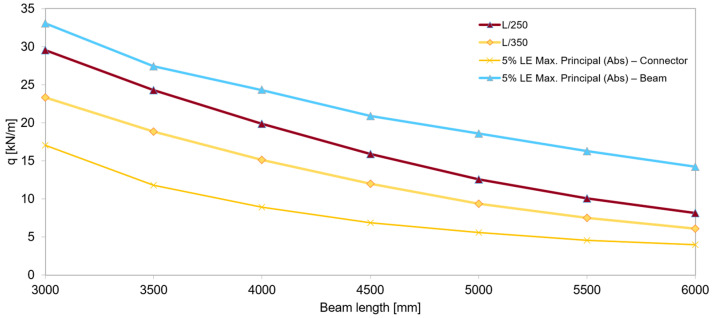
Load-bearing capacity of the central perforated beam for different spans and load-bearing criteria.

Figure 31 presents the load-bearing capacity curves for the central perforated thin-walled beam spanning from 3 to 6 m. This beam functions as part of a structural system incorporating a chipboard deck, which interacts with the beam to provide composite action and enhance overall structural performance. The curves illustrate how load-bearing behavior varies with span length and loading conditions, offering insight into the system’s response and limitations.

Based on the results of detailed numerical simulations, four distinct load-bearing criteria were established to assess the system’s performance. These criteria address both serviceability and ultimate limit states, ensuring a comprehensive evaluation of structural behavior:

1. L/250—This criterion corresponds to the allowable deflection relative to the beam span and is commonly used in general construction as a serviceability limit to ensure acceptable deformation levels.
2. L/350—A more conservative deflection limit than L/250, applied in cases requiring increased stiffness and stricter serviceability conditions, such as in structures with sensitive finishes or precision equipment.
3. 5%. LE Max. Principal (Abs) in the connector—This limit indicates the onset of plastic deformation in the connector, marking a critical stage at which the component begins to yield and lose its elastic behavior under load.
4. 5%. LE Max. Principal (Abs) in the beam—This criterion defines the point at which the beam itself begins to plastify, signaling a transition from elastic to plastic behavior and providing a reference for ultimate load-bearing capacity.

Together, these criteria form a robust framework for evaluating the structural integrity and performance of the beam–chipboard system across different configurations and span lengths.

## 4. Conclusions

This study presented validated numerical simulations of a thin-walled perforated beam cooperating with a P4-class chipboard deck, aiming to determine load-bearing capacity curves over spans from 3 to 6 m and to investigate associated failure mechanisms.

Experimental validation confirmed the accuracy of the model, with good agreement in load–displacement behavior, deformation modes, and failure progression. The numerical model proved reliable for assessing global and local structural responses in the elastic–plastic range.

The key findings are summarized below:

- Failure mechanisms depend on span length. In 3 m beams, plasticization in the central zone occurred nearly simultaneously with deck panel fracture, indicating strong composite interaction. For 6 m spans, deck fracture preceded beam yielding, reflecting reduced structural synergy due to higher deflections and stress.
- Consistent results across simulations and experiments. The model accurately reproduced the initiation and development of failure, confirming its predictive capability.
- Horizontal displacement patterns. Significant lateral displacements occurred consistently in the lower flange, as the upper flange was stiffened by the chipboard. To mitigate this, additional reinforcement or bracing between the bottom flanges is recommended, although not yet modeled.
- Identification of critical elements. The connectors and support zones were found to be the weakest components. In all configurations, failure began with connector plasticization, regardless of span.

In summary, this research offers a novel contribution by developing a validated finite element model that accurately captures the interaction between perforated thin-walled beams and chipboard panels, a combination rarely addressed in the literature. Unlike previous studies focused solely on perforated beams or beams interacting with steel gratings, our work covers the full span range (3 to 6 m) and introduces detailed load-bearing capacity curves based on multiple failure criteria, including connector yielding and mid-span beam plasticization. Moreover, the study identifies connectors as the critical failure points across different configurations, providing valuable insights for the design and optimization of such composite systems. These findings not only improve structural understanding but also offer practical guidance for enhancing the safety and efficiency of lightweight structural systems incorporating perforated profiles and wood-based materials.

## Figures and Tables

**Figure 23:**
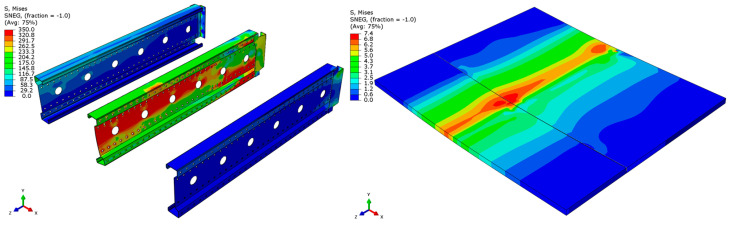
Deformation and von Mises stresses for 3 m beams.

**Figure 24:**
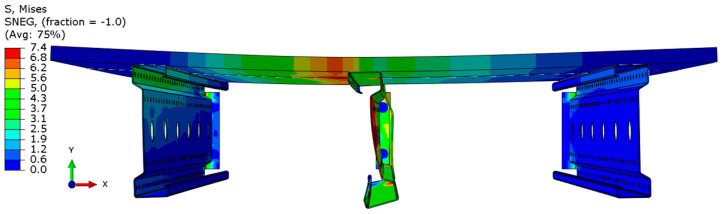
Deformation and von Mises stresses for 3 m beams—cross-section.

**Figure 25:**
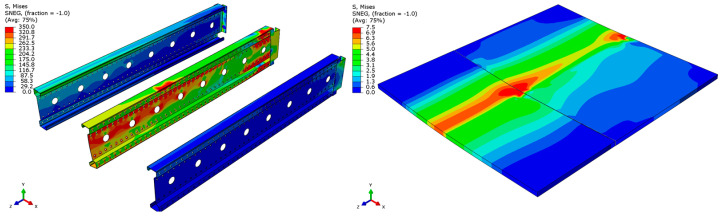
Deformation and von Mises stresses for 4 m beams.

**Figure 26:**
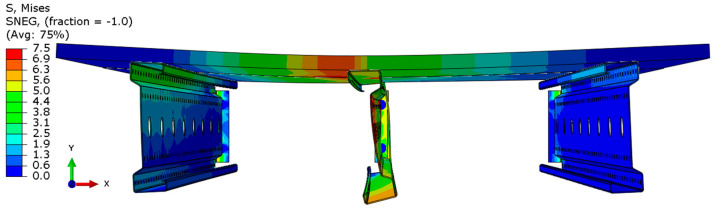
Deformation and von Mises stresses for 4 m beams—cross-section.

**Figure 27:**
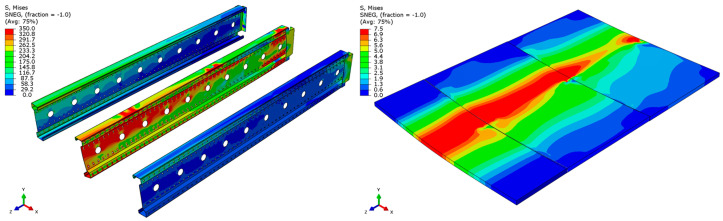
Deformation and von Mises stresses for 5 m beams.

**Figure 28:**
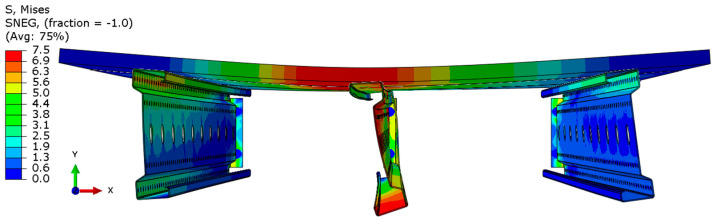
Deformation and von Mises stresses for 5 m beams—cross-section.

**Figure 29:**
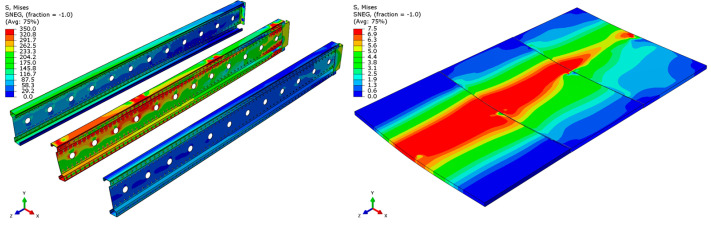
Deformation and von Mises stresses for 6 m beams.

**Figure 30:**
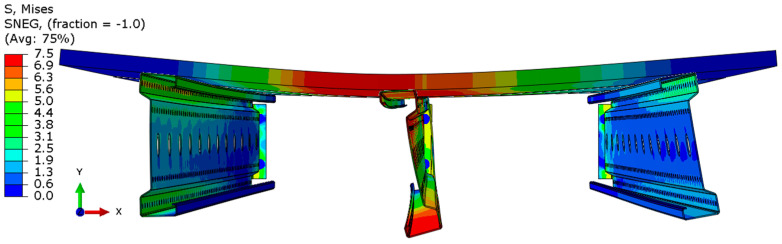
Deformation and von Mises stresses for 6 m beams—cross-section.

**Table 1:** Percentage of load carried by individual beams based on deflection comparison.

Length of the Beam [m]	Load Taken over by the Left Beam [%]	Load Taken over by the Central Beam [%]	Load Taken over by the Right Beam [%]
3.0	10.406	83.225	6.369
3.5	11.201	81.190	7.609
4.0	12.524	78.412	9.064
4.5	14.364	74.540	11.096
5.0	15.370	70.599	14.031
5.5	16.074	65.765	18.161
6.0	18.326	61.501	20.174

**Table 2:** Comparison of experimental and numerical results.

Configuration	Max Load (Experiment) [kN]	Max Load (Simulation) [kN]	Difference [%]
3 m (P6 deck)	103.8	108.1	4.14
6 m (P6 deck)	72.7	77.1	6.05
6 m (P4 deck)	61.2	62.7	2.45

## Data Availability

The original contributions presented in the study are included in the article, further inquiries can be directed to the corresponding author.
